# The effects of silver nanoparticles on antimicrobial activity of ProRoot mineral trioxide aggregate (MTA) and calcium enriched mixture (CEM)

**DOI:** 10.4317/jced.52568

**Published:** 2016-02-01

**Authors:** Nematollah Jonaidi-Jafari, Morteza Izadi, Pedram Javidi

**Affiliations:** 1 Health Research Center, Baqiyatallah University of Medical Sciences, Tehran, IR Iran; 2 Department of Orthodontics, Faculty of Dentistry, Ahvaz Jundishapur University of Medical Sciences, Ahvaz, IR Iran

## Abstract

**Background:**

Although, mineral trioxide aggregate (MTA) and new experimental cement (CEM) are good root filling cements, but had no or low antimicrobial activities. The aim of this study was to evaluate the effects of addition of silver nanoparticles (SNP) to these two cements on antimicrobial effects against five most dental infection related microorganisms.

**Material and Methods:**

Two suspensions of 100 and 200 ppm of SNP were prepared and 180 μl of microbial suspension with 1.5 × 108 CFU/ml of each respected microorganisms were re-suspended in deionized water or each of SNP suspensions. After that, 60 μg of MTA and CEM were added to each tube. In one tube, the mixture of all above mentioned microorganisms were added as a source of microorganism. Colonies were counted after 0, 24, 48, 72 and 96 hours intervals of incubation at 35°C on blood agar for evaluation of antimicrobial efficacy.

**Results:**

MTA and CEM had antibacterial activities on all microorganisms’ strains except for Enterococcus faecalis and mixture group. MTA had better antibacterial activity than CEM but the difference was not significant (*p*<0.05). The combination of SNP with two cements resulted in significantly higher antimicrobial activities (*p*<0.05). Also, there was no statistically significant difference between two SNP concentrations (*p*>0.05).

**Conclusions:**

Mixture of MTA and CEM with different concentrations of SNP significantly increased the antibacterial activity.

** Key words:**Mineral trioxide aggregate, calcium-enriched mixture, silver nanoparticle, antimicrobial activity.

## Introduction

Root-end fillings are able to seal the content of a root canal system. This sealing prevents egress of microorganisms or byproducts into periradicular tissues (1,2). Some examples of the existing root-end-filling materials are gutta-percha, zinc oxide eugenol-based cements (i.e. Super-EBA and IRM), composite resin, glass ionomer cement, Cavit, gold foil, polycarboxylate cement, polyvinyl cement, amalgam, Vitremer, and mineral trioxide aggregate (MTA) (1,3). MTA is currently marketed in 2 forms: grey (GMTA) and white (WMTA) (3). It was introduced in grey, but because of the discoloration potential of GMTA, WMTA was developed (2-6). Several studies examined the antibacterial effects of MTA and its variants on various organisms and showed beneficial effects with some conflicting reports. In many studies reported that MTA has limited antimicrobial effect against some microorganisms (2,6-8), however, others reported antimicrobial activity of MTA, especially in grey form, on *Micrococcus luteus, Staphylococcus aureus, E.coli, Pseudomonas aeruginosa, Candida albicans*, and *Enterococcus faecalis* (9,10).

Recently, new experimental cement; Calcium Enriched Mixture (CEM) consisting of different calcium compounds (e.g., calcium oxide, calcium phosphate, calcium carbonate, calcium silicate, calcium sulfate, calcium hydroxide and calcium chloride) has been developed (11). The clinical applications of CEM are similar to those of MTA, and both cements have a similar working time, pH and dimensional stability (11-16). Most efforts were done to improve the antimicrobial effects of both cements and among them, using of new materials such as silver nanoparticle (SNP) takes more attention. Although, it has been demonstrated that SNP is a good antimicrobial agent but, reports about using of SNP with MTA and CEM and comparison of their antibacterial and antifungal effects are scarce (17-19). Therefore, the aim of this study was to evaluate the effects of SNP with different concentrations on antimicrobial activity of ProRoot MTA and CEM mixed against the most five important microorganism species.

## Material and Methods

-Microorganisms

The study was conducted under climate-controlled conditions (23 ± 2°C; 50 ± 10% relative air humidity). In this study five microorganism species were used to evaluate the antimicrobial effects of MTA and CEM. The test materials, MTA (Dentsply, Tulsa dental, OK, USA) and CEM (Shahid Beheshti University, Tehran, Iran), were manipulated strictly in accordance with the manufacturer’s instructions. Three standard bacterial strains include *Escherichia coli* (ATCC 29929), *Actinomyces* (ATCC 15987) and *Streptococcus mutans* (ATCC 25157). *Candida albicans* and *Enterococcus faecalis* isolated by the Central Microbiological Laboratory (Imam Reza Hospital, Mashhad, Iran) also was included in the study. Microbial strains were confirmed by both Gram staining and colony forming and growth characteristics.

-SNP preparation

The SNP were synthesized at School of Pharmacy, Mashhad University of Medical Sciences, Mashhad, Iran and their diameter and spectral properties were evaluated according to the previous reports (20-22). Briefly, the spectral properties of our formulated SNP were checked in the spectrum of 200-800 nm with spectrophotometer. Also, the mean SNP diameter was reported as 70 nm by using the particle size analyzer.

-Cement preparation and antimicrobial assay

Two suspensions of 100 and 200 ppm of the SNP were prepared. 180 μl of suspension with 1.5 × 108 CFU/ml of each respected microorganisms were re-suspend in deionized water or each of SNP suspensions and then, 60 μg of MTA and CEM were added to each tube. In one tube, the mixture of all above mentioned microorganisms was added as source of microorganism. One negative control (without microorganisms) and one positive control without two cements also were prepared. Colonies were counted after 0, 24, 48, 72 and 96 hours intervals of incubation at 35°C on blood agar for the evaluation of antimicrobial efficacy. Colony counts were done by Standard Plate method. In this method cell count is done by diluting the original sample, plating dilutions onto a culture medium and then after incubation under proper conditions, the total number of viable cells is reported as colony forming units (CFUs).

-Statistical analysis

All data were expressed as mean ± SD and were analyzed using two independent sample T test and one way ANOVA by SPSS version 16. The *P* value lower than 0.05 was considered as significant difference.

## Results

**Figure 1:**
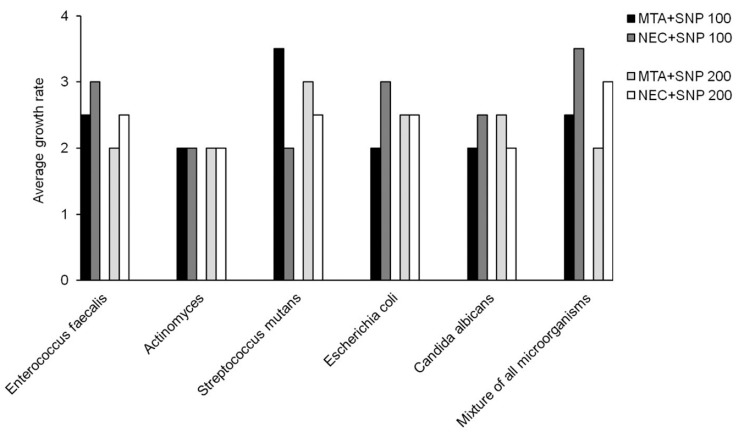
Average growth rate of different microbial strains in mixture of mineral trioxide aggregate (MTA) and new experimental cement (CEM) with two concentrations of silver nanoparticle (SNP).

Frequency and percentage of grown microorganisms in aqueous and SNP suspensions of MTA and CEM were presented in  Table 1. Mann-Whitney analysis demonstrated that although in both aqueous and SNP suspensions, the antimicrobial activity of MTA was higher than CEM, but these differences were not significant (*p*=0.13 and *p*=0.63, respectively). The average growth rates of microorganisms in different times of culture based on different cements were presented in  Table 2. Our analysis demonstrated that MTA at 0 and 96 hours had the greater antimicrobial activity than CEM and also with increasing the time of culture, the antimicrobial activities of both MTA and CEM were increased. However, none of them were significant (*p*>0.05). The average growth rate of each microorganism in MTA and CEM are presented in  Table 3. These data demonstrated that neither MTA nor CEM had antibacterial effects against *E. faecalis* and mixture of all microorganisms. Also, the most antimicrobial effects of MTA and CEM were against *C. albicans* and Actinomyces, respectively. However, Mann-Whitney analysis revealed that there was significant difference in antimicrobial effects of MTA and CEM only against *C. albicans* (*p*=0.003). Antimicrobial effects of different suspensions of MTA and CEM were compared ant presented in  Table 4. Kruskal-Wallis analysis demonstrated that using of SNP in both concentrations in combination with MTA and CEM could increase their antimicrobial effects significantly against all tested microorganisms (*p*<0.05). However, as demonstrated in figure 1, Mann-Whitney analysis revealed that there was no significant difference between different SNP concentrations (*p*>0.05).

**Table 1:**
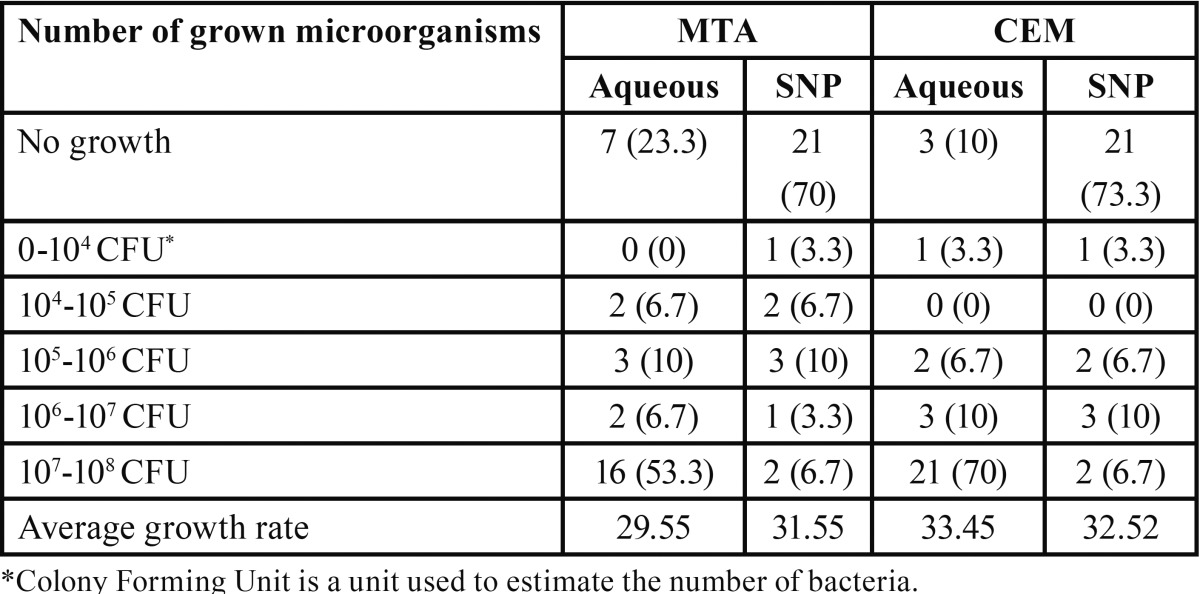
Frequency (percentage) of grown microorganisms on aqueous and silver nanoparticle suspension of MTA and CEM.

**Table 2:**
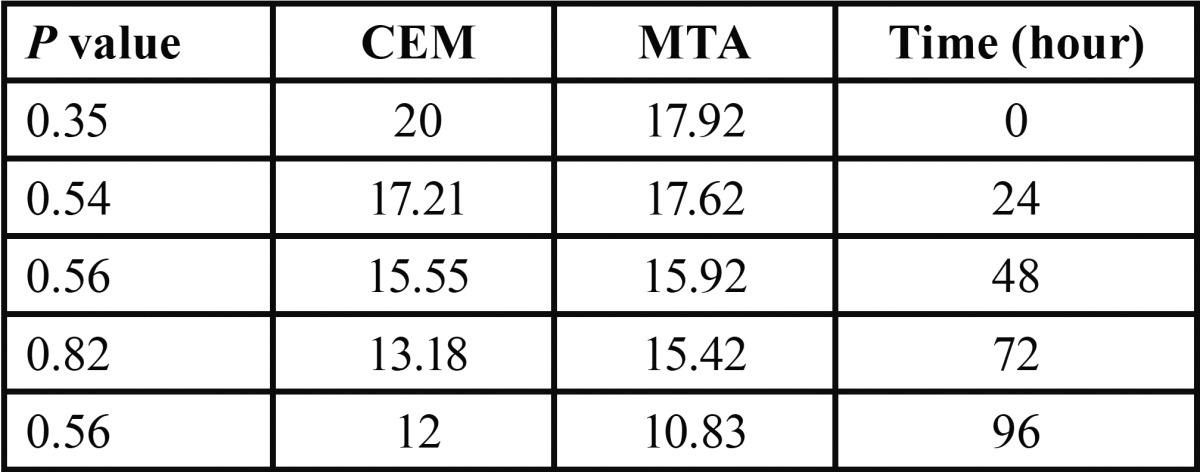
The average growth rate in different times according to the type of cement.

**Table 3:**
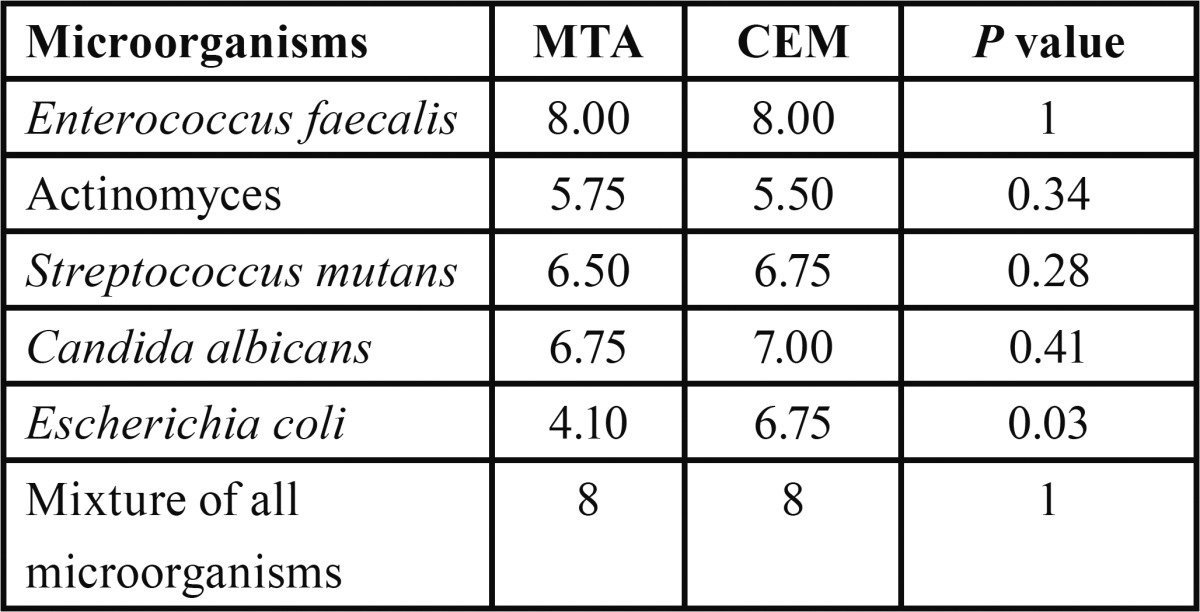
Average growth rate of different microorganisms in MTA and CEM.

**Table 4:**
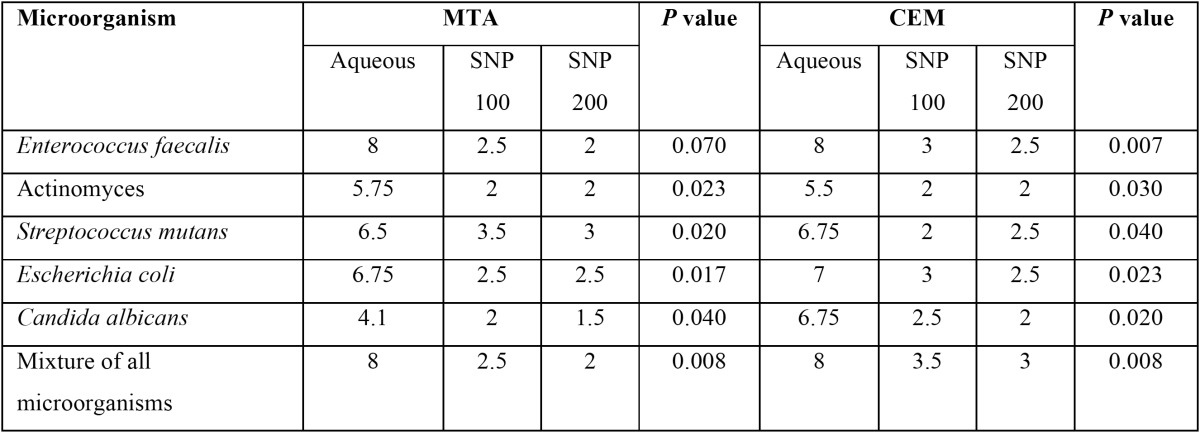
Comparison of average bacterial growth rates in different suspensions of MTA and CEM.

## Discussion

The most important cause of apical periodontitis is inflamed or necrotic pulp, which is produced by colonization of microorganisms and even can lead to bone infection. Therefore, antibacterial properties of materials and cements used in root canal treatments are important (21,22). In this study, the effects of SNP on the antimicrobial effects of two cements including MTA and CEM were evaluated. Our results demonstrated that mixture of MTA and CEM with different concentrations of SNP significantly increased their antimicrobial activity. It has been demonstrated that both MTA and CEM showed some antimicrobial effects, however this efficacy is not sufficient. In all dental research focusing on the antimicrobial agents, *E. feacalis* was used as reference microorganism, because it is a Gram-positive bacterial infection often found in drug resistant apical periodontitis and its elimination is often challenging (4). Our enrolled microorganisms include *E. faecalis*, *E. coli* as the reference microorganism (22), Actinomyces as resistant microorganism (16) found in secondary infection of root canal and *C. albicans* as the most common root canal cases of treatment failure (14,23,24).

There is a lack of consensus about the antimicrobial effects of MTA and CEM between researchers. Torabinejad *et al.* (25) (1995) reported that Loma Linda MTA could not inhibit the growth of *E. faecalis*, *S. aurous* and *Fusobacterium nucleatum*. However, Stowe *et al.* (26) (2004) in their study with quite opposite results, reported that aqueous suspension of MTA could inhibit the growth rate of all of these three microorganisms (17). Lack of antibacterial effects against *E. faecalis* had been reported for MTA (16,26) and CEM (16). Our results about antibacterial effects of MTA and CEM in aqueous form are in line with studies reported by Zarrabi *et al.* (27) (2009) and Eldeniz *et al.* (6) (2006) and demonstrated that these two cements in this form could not inhibit the growth of *E. faecalis*. The main possible cause of this observation is the ability of *E. faecalis* in changing of its cell wall structure. This changing also increases its resistance against high pH and temperature (27). However, using of SNP can significantly affect their antibacterial effects against *E. faecalis* and other evaluated microorganisms. Our results about antifungal properties of MTA and CEM are in agreement with previous report (17) and demonstrated that MTA had the most efficacies against *C. albicans*. The proposed mechanism for antibacterial and antifungal properties of MTA and CEM is the release of calcium hydroxide. This ion can increase the environmental pH and therefore, it makes the surrounding conditions inappropriate for microorganisms (13). However, the main cause of the observed differences in the various studies can be due to the different methods for assessing of antimicrobial effects including agar diffusion and contact dilution.

The addition of silver and zinc to heat polymerized acrylic resins is consistent with the current trend of incorporating antimicrobials into dental materials (20). Also, the antibacterial activity of six types of nano-silver base inorganic antibacterial agents on oral pathogens were assessed and compared. The results of this study demonstrated that nano-silver base inorganic antibacterial agents had fine bactericidal activity against oral pathogens and it is possible that nano-silver base inorganic antibacterial agents can be used in dental antibacterial materials (10). Also, *in vitro* evaluation of antimicrobial effect of silver-zeolite on *C. albicans* and nosocomial respiratory infection-causing bacteria, *S. aureus* and *P. aeruginosa* demonstrated that silver-zeolite had antimicrobial effects for four weeks on *C. albicans* and nosocomial respiratory infection-causing bacteria in saliva *in vitro* (7). Our results about the positive effects of SNP on MTA and CEM antimicrobial activities are in agreement with other previous reports and also demonstrated that there were no significant differences between two SNP concentrations. Silver particle can decrease the attachment of microorganisms to the surface (9) and also increase the antibacterial properties of endodontic sealers (12).

Within the limitation of this preliminary study, it may be concluded that the addition of low percentages of silver nanoparticle to MTA and CEM can be a valuable alternative for increasing antimicrobial effects of such materials. However, conducting of further studies on the mechanism of positive effects of SNP on the antimicrobial effects of endodontic cement is highly recommended.

## References

[1] Al-Nazhan S, Al-Judai A (2003). Evaluation of antifungal activity of mineral trioxide aggregate. J Endod.

[2] Asgary S, Shahabi S, Jafarzadeh T, Amini S, Kheirieh S (2008). The properties of a new endodontic material. J Endod.

[3] Basrani B, Tjaderhane L, Santos JM, Pascon E, Grad H, Lawrence HP (2003). Efficacy of chlorhexidine- and calcium hydroxide-containing medicaments against Enterococcus faecalis in vitro. Oral Surg Oral Med Oral Pathol Oral Radiol Endod.

[4] Xavier CB, Weismann R, de Oliveira MG, Demarco FF, Pozza DH (2005). Root-end filling materials: apical microleakage and marginal adaptation. J Endod.

[5] Casemiro LA, Gomes Martins CH, Pires-de-Souza Fde C, Panzeri H (2008). Antimicrobial and mechanical properties of acrylic resins with incorporated silver-zinc zeolite - part I. Gerodontology.

[6] Eldeniz AU, Hadimli HH, Ataoglu H, Orstavik D (2006). Antibacterial effect of selected root-end filling materials. J Endod.

[7] Estrela C, Bammann LL, Estrela CR, Silva RS, Pecora JD (2000). Antimicrobial and chemical study of MTA, Portland cement, calcium hydroxide paste, Sealapex and Dycal. Braz Dent J.

[8] Fridland M, Rosado R (2005). MTA solubility: a long term study. J Endod.

[9] Gartner AH, Dorn SO (1992). Advances in endodontic surgery. Dent Clin North Am.

[10] Gomes-Filho JE, Silva FO, Watanabe S, Cintra LT, Tendoro KV, Dalto LG (2010). Tissue reaction to silver nanoparticles dispersion as an alternative irrigating solution. J Endod.

[11] Jenkinson HF (1994). Cell surface protein receptors in oral streptococci. FEMS Microbiol Lett.

[12] Kim S, Choi JE, Choi J, Chung KH, Park K, Yi J (2009). Oxidative stress-dependent toxicity of silver nanoparticles in human hepatoma cells. Toxicol In Vitro.

[13] Kishen A, Shi Z, Shrestha A, Neoh KG (2008). An investigation on the antibacterial and antibiofilm efficacy of cationic nanoparticulates for root canal disinfection. J Endod.

[14] Kratchman SI (2004). Perforation repair and one-step apexification procedures. Dent Clin North Am.

[15] Matsuura T, Abe Y, Sato Y, Okamoto K, Ueshige M, Akagawa Y (1997). Prolonged antimicrobial effect of tissue conditioners containing silver-zeolite. J Dent.

[16] Miyagak DC, de Carvalho EM, Robazza CR, Chavasco JK, Levorato GL (2006). In vitro evaluation of the antimicrobial activity of endodontic sealers. Braz Oral Res.

[17] Mohammadi Z, Modaresi J, Yazdizadeh M (2006). Evaluation of the antifungal effects of mineral trioxide aggregate materials. Aust Endod J.

[18] Parirokh M, Torabinejad M (2010). Mineral trioxide aggregate: a comprehensive literature review--part I: chemical, physical, and antibacterial properties. J Endod.

[19] Schäfer E, Bössmann K (2005). Antimicrobial efficacy of chlorhexidine and two calcium hydroxide formulations against Enterococcus faecalis. J Endod.

[20] She WJ, Zhang FQ (2003). Comparison of the antibacterial activity on oral pathogens among six types of nano-silver base inorganic antibacterial agents. Shanghai Kou Qiang Yi Xue.

[21] Shrestha A, Shi Z, Neoh KG, Kishen A (2010). Nanoparticulates for antibiofilm treatment and effect of aging on its antibacterial activity. J Endod.

[22] Sondi I, Salopek-Sondi B (2004). Silver nanoparticles as antimicrobial agent: a case study on E. coli as a model for Gram-negative bacteria. J Colloid Interface Sci.

[23] Yasuda Y, Kamaguchi A, Saito T (2008). In vitro evaluation of the antimicrobial activity of a new resin-based endodontic sealer against endodontic pathogens. J Oral Sci.

[24] Tanomaru-Filho M, Tanomaru JM, Barros DB, Watanabe E, Ito IY (2007). In vitro antimicrobial activity of endodontic sealers, MTA-based cements and Portland cement. J Oral Sci.

[25] Torabinejad M, Hong CU, Pitt Ford TR, Kettering JD (1995). Antibacterial effects of some root end filling materials. J Endod.

[26] Stowe TJ, Sedgley CM, Stowe B, Fenno JC (2004). The effects of chlorhexidine gluconate (0.12%) on the antimicrobial properties of tooth-colored ProRoot mineral trioxide aggregate. J Endod.

[27] Hasan Zarrabi M, Javidi M, Naderinasab M, Gharechahi M (2009). Comparative evaluation of antimicrobial activity of three cements: new endodontic cement (NEC), mineral trioxide aggregate (MTA) and Portland. J Oral Sci.

